# Bacterial Communities in Sand and Seawater of Northern Gulf Coast Beaches: Temporal, Spatial, and Environmental Influences

**DOI:** 10.1111/1758-2229.70309

**Published:** 2026-03-02

**Authors:** Stephanie N. Vaughn, Jacqueline C. Pavlovsky, Colin R. Jackson

**Affiliations:** ^1^ Department of Biology University of Mississippi University Mississippi USA

**Keywords:** bacterial diversity, coastal ecology, sand microbiome, seawater microbiome, spatiotemporal patterns

## Abstract

Coastal microbial communities play critical roles in marine food webs and biogeochemical cycling, yet their diversity and function remain poorly characterised in many regions. This is especially evident along the northern Gulf coast, a dynamic system with substantial freshwater influences. We used high throughput 16S rRNA sequencing to characterise bacterial communities in sand and seawater collected every 3 months (March 2024 through March 2025) from 10 beaches along a 53 km stretch of the Mississippi coast. The diversity and composition of these communities were related to environmental variation and to biogeochemical function as determined from the activity of enzymes related to carbon, nitrogen, and phosphorus mineralisation. Our findings revealed distinct bacterial communities in sand and seawater, with the microbiome of each habitat showing greater temporal variation over the course of the study than spatial variation between beaches. Patterns in bacterial community structure and proportions of abundant taxa were strongly linked to physicochemical variables, while enzyme activities suggested how microbial communities may contribute to biogeochemical processes in these habitats. Collectively, these findings provide critical information for understanding microbial ecology in this system and highlight the central role of bacteria in mediating ecosystem function along a dynamic and understudied coastline.

## Graphical abstract

Bacterial communities in sand and seawater collected every 3 months from 10 beaches along the Mississippi Gulf coast were fundamentally different from each other. Bacterial communities in both habitats showed more temporal variation over the course of 15 months than spatial variation between beaches.

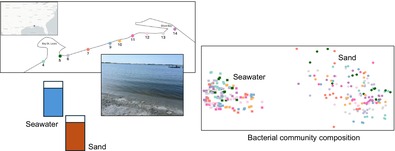

## 1 Introduction

Marine environments are shaped by physical and chemical factors which fluctuate in response to natural and anthropogenic events (Biasutti et al. 2012; Doney et al. 2012; Fischer et al. 2014; Prakash 2021). In the northern Gulf of Mexico, nutrient‐rich inputs of freshwater can cause extensive algal blooms and hypoxic areas such as the Gulf Dead Zone (Ainsworth et al. 2015; Cambazoglu et al. 2017; Diaz and Rosenberg 2008; Jenny et al. 2016). While much attention has focused on inputs from the Mississippi River, the northern Gulf coast also faces increasing inputs of freshwater from other sources, as well as rising concentrations of chlorophyll, fluctuating salinity, and increasing water temperature (Biasutti et al. 2012; Frolova and Miglietta 2020). Despite being a region of substantial biodiversity and supporting a significant amount of fish and shellfish production (Ainsworth et al. 2015; Cambazoglu et al. 2017; Karnauskas et al. 2015; Kiskaddon et al. 2022; Moragoda et al. 2024), the northern Gulf coast has been understudied in terms of its ecology, especially of microorganisms. There is a need to understand how environmental conditions influence the microbial communities in this system, as microbial communities form the foundation of marine food webs and can have major impacts on ecosystem health (Fortunato and Crump 2011; Fuhrman et al. 2015; Rhodes et al. 2024; Sunagawa et al. 2015). Comprehensive studies on bacterial communities of the northern Gulf coast are limited, with only minimal characterization of this system's bacterial diversity and function.

Microorganisms are important to the biogeochemistry of marine systems by processing organic matter, nutrients, and pollutants (Bunse and Pinhassi 2017; Fuhrman et al. 2015; Rhodes et al. 2024; Wang et al. 2019). Almost half of the global biogeochemical fluxes of carbon, nitrogen, phosphorus, sulfur, and iron are processed through the metabolic pathways of microorganisms (Bunse and Pinhassi 2017; Fuhrman et al. 2015), with different microbial groups playing roles that are influenced by water quality and environmental conditions (Bunse and Pinhassi 2017; Wang et al. 2019; Xu et al. 2021). These microorganisms are important for higher trophic levels and understanding patterns in bacterial community composition over space and time is critical to our knowledge of marine food webs (Bunse and Pinhassi 2017; Lucas et al. 2015; Spietz et al. 2015; Ward et al. 2017). Research in the Chesapeake Bay (USA) found temporal patterns in marine bacterioplankton communities, but spatial patterns were only apparent in individual seasons (Wang et al. 2020). Similarly, bacterioplankton communities off the North Carolina coast (USA) showed strong annual patterns, with spatial patterns in community structure being dependent on distance from the coast (Wang et al. 2019; Ward et al. 2017). Examining these and other bacterial communities in coastal ecosystems may be particularly important, as the nearshore environment is influenced by both the land and ocean (Rhodes et al. 2024), especially in areas with a large freshwater influence, such as the Mississippi coast.

The Mississippi coast borders the Mississippi Sound, a stretch of water that is separated from the rest of the Gulf of Mexico by barrier islands and sandbars (Cambazoglu et al. 2017; Dzwonkowski et al. 2018). While several smaller rivers discharge water to the Mississippi coast, it can be impacted most dramatically by occasional freshwater diversions through the Bonnet Carré Spillway, a flood‐protection system that releases large amounts of nutrient‐rich water from the Mississippi River into Lake Pontchartrain and eventually the Mississippi Sound. Such diversions are rare, the last being 2019, but lead to substantial changes in salinity, water quality, and marine communities (Gledhill et al. 2020; Moody et al. 2024; Soto Ramos et al. 2023). Overall, however, the Mississippi coast has been understudied ecologically, particularly in terms of its microbial communities. This study provides the first comprehensive analysis of bacterial communities in seawater and sand from nearshore locations (beaches) along the Mississippi coast. We examine how the richness and diversity of these communities vary across seasons, over spatial distances of > 50 km, and in relation to physicochemical conditions. Our findings are important for understanding bacterial community structure along the beaches of this important part of the northern Gulf of Mexico and provide a baseline for future investigations in similar marine systems.

## 2 Experimental Procedures

### 2.1 Sample Collection and Processing

**FIGURE 1:**
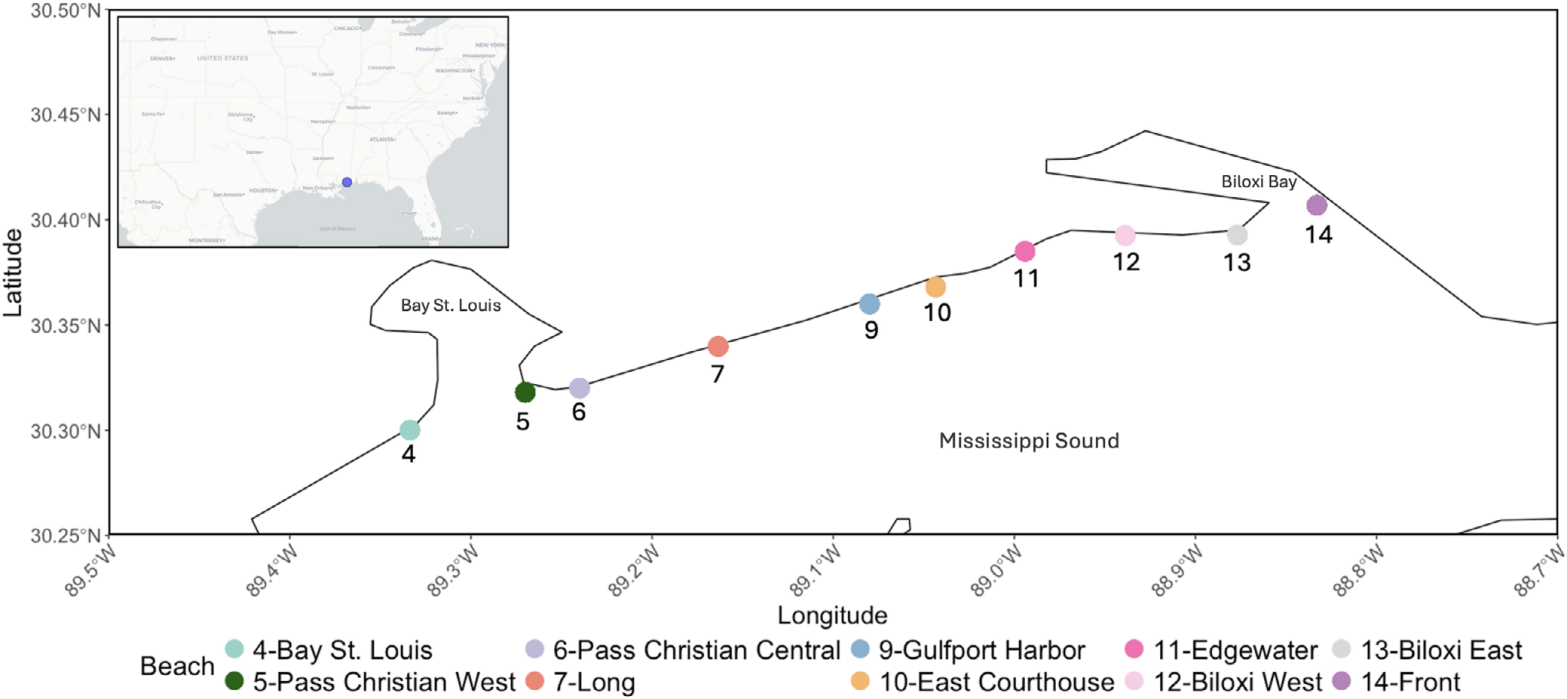
Coordinates (decimal degrees) of 10 beaches spanning 53.1 km along the Mississippi coast, sampled every 3 months from March 2024 to March 2025 to characterise the bacterial communities of seawater and sand. Beach numbers correspond to designations used by the Mississippi Department of Environmental Quality, which conducts public health related monitoring of these beaches, and are shown along with beach names.

Seawater and sand were collected from 10 beaches along the Mississippi coast every 3 months for a year, starting in March 2024 and ending in March 2025 (i.e., sampling in March, June, September, December, and March). The beaches sampled spanned 53.1 km along the coast, with an average of 6.3 km between beaches, and were identified with site numbers corresponding to the Mississippi Department of Environmental Quality (MDEQ) Beach Monitoring Program (Figure 1), which conducts ongoing public health related monitoring of these beaches.

Sampling on each date began at the western most beach (MDEQ site 4, Bay St. Louis Beach) at approximately 11:00 and ended at the eastern most beach (MDEQ site 14, Front Beach) at or before 14:00. This region only experiences one high and one low tide per day, with minimal (typically < 0.6 m) variation in tidal depth, such that the maximum 3‐h difference between the first and last sample collected was unlikely to be influenced by tidal variation on a given sampling date. Seawater and sand were collected from the shoreline‐water interface of each beach, where seawater was overlying and inundating sand. Three independent replicate samples of each of seawater and sand were collected < 1 m apart from each beach on each sample date (i.e., 30 samples of seawater and 30 samples of sand collected on each date, for a total of 300 samples over the course of the study). Seawater samples were collected directly into sterile 500 mL Nalgene bottles from surface water at approximately 0–0.3 m depth that was being mixed by gentle tidal action. Sand samples were collected from the surf zone by scooping moist surface sand into sterile 50 mL centrifuge tubes close to the area of seawater collection. Water temperature (°C), pH, dissolved oxygen (DO; %), and salinity (ppt) were measured in the water at each beach using handheld probes, and air temperature (°C), and GPS coordinates were recorded. Samples were kept on ice and transported to the University of Mississippi (UM) for processing within 18 h.

For seawater, 200 mL of each sample was filtered through a sterile 0.2 μm nylon membrane filter (MilliporeSigma, Cork, Ireland), which was then cut in half. One half of the filter was transferred into a bead tube from a PowerSoil Pro DNA extraction kit (Qiagen, Germantown, MD, USA), while the other half was archived. For sand, 0.5 g of each sample was placed into a bead tube for DNA extraction, with the remaining portion of each sand sample archived. Samples for DNA extraction and archiving were stored at −20°C until further processing.

To examine connections between bacterial community structure and biogeochemical processes, assays of extracellular enzyme activity of sand and unfiltered seawater were conducted in unison with sample processing and conducted within 24 h of sample collection. Enzyme assays followed the protocols of Jackson et al. (2013) and Basha et al. (2025) and used 4‐methylumbelliferone (MUB) linked fluorogenic substrates to measure activity in seawater and para‐nitrophenyl (pNP) linked colorimetric substrates to measure activity in sand. Activity was measured for enzymes involved in the degradation of organic carbon (β‐glucosidase), phosphate (phosphatase), and carbon and nitrogen (N‐acetylglucosaminidase; NAGase). Sand was also assayed for the activity of the oxidative enzymes phenol oxidase and peroxidase using L‐dihydroxyphenylalanine (L‐DOPA) as a substrate (protocol based on Sinsabaugh et al. 2005 and DeForest 2009). All activities were expressed as nmoles of substrate consumed h^−1^ L^−1^ of seawater or nmoles of substrate consumed h^−1^ g^−1^ dry mass of sand.

### 2.2 DNA Extraction, Amplification, and Sequencing

DNA was extracted from filters and sand using a PowerSoil Pro DNA extraction kit, following the manufacturer's protocol, and DNA was visualised by agarose gel electrophoresis. A dual‐index barcoding approach was used for Illumina next‐generation sequencing (Kozich et al. 2013). Procedures followed those described by previous studies (Jackson et al. 2015; Vaughn et al. 2024), where DNA was amplified targeting 250 base pairs (bp) of the V4 region of the bacterial 16S rRNA gene (Kozich et al. 2013). Each primer was tagged with a specific 8‐nucleotide barcode to allow subsequent pooling of samples. Following amplification, barcoded amplicons were standardised using SequalPrep plates (Life Technologies, Grand Island, NY, USA) and pooled prior to sequencing. The assembled library was spiked with 20% PhiX (Jackson et al. 2015) and sequenced using an Illumina NextSeq at the University of Mississippi Medical Center (UMMC) Molecular and Genomics Core Facility.

Raw sequence files (fastq) were downloaded and processed using the standard 16S rRNA pipeline of the DADA2 package version 1.26.0, within R version 4.2.2. Sequences were trimmed at truncLen = c(240,190) and quality profile plots inspected to ensure proper quality of trimmed reads. Forward and reverse reads were merged with minLen = 135 bp, and sequences < 250 or > 256 bp were removed, as were sequences identified as potential chimeras, chloroplasts, mitochondria, Archaea, or Eukarya. Unique sequences were inferred as amplicon sequence variants (ASVs) and taxonomy was assigned to ASVs using the SILVA database (Quast et al. 2013) v138.1 (released August 2020) with nomenclature of phyla following Oren and Garrity (2021). Prior to downstream community analysis, singleton ASVs were removed and samples were rarefied by randomly subsampling each to the lowest sequence count observed across all samples.

### 2.3 Statistical Analyses

A map of the Mississippi Coast (Figure 1) was generated in R using the packages ‘rnaturalearth’ (Massicotte and South 2023), ‘rnaturalearthdata’ (South et al. 2024) ‘sf’ (Pebesma and Bivand 2023), and ‘ggplot2’ (Wickham 2016). Coastline shapefiles were obtained from Natural Earth data and cropped to the extent of the study region. The coordinates from beaches sampled in this study were converted to spatial features (WGS84 coordinate system) and overlaid on the cropped coastline. Subsequent maps followed the same methodology while displaying environmental parameters and bacterial community characteristics. Analysis of variance (ANOVA) tests were used to determine differences in each environmental parameter measured (temperature, pH, DO, salinity) between sampling dates and beaches. Partial Mantel correlations, using Spearman's correlations co‐efficient, were used to determine the relationship between each environmental parameter and temporal distance (days) while controlling for geographic distance (km) between beaches, and vice versa. Temporal distances were calculated as the number of days separating sampling dates, and geographic distances (km) between beaches were calculated using great‐circle (Haversine) distances based on recorded latitude and longitude coordinates using the ‘geosphere’ package (Hijmans 2022).

After clustering of sequences into ASVs, all samples were rarefied to 5406 sequences (the lowest sequence count observed across all samples) using the *Rarefy* function of the ‘GUniFrac’ package in R (Chen et al. 2012). Good's coverage scores were calculated using the phyloseq_coverage function of the metagMisc R package (Mikryukov 2017) to determine the effective sequencing depth of each sample. Permutational analysis of variance (PERMANOVA) was used to test for significant differences in Bray–Curtis dissimilarity among samples across sample type (i.e., seawater/sand), sampling date, and beach. Homogeneity of variance between each categorical factor was analysed using *permutest* and *betadisper* in ‘vegan’ (Oksanen et al. 2022). Non‐metric multidimensional scaling plots (NMDS) were used to distribute Bray–Curtis dissimilarity scores of samples to visualise spatiotemporal differences in bacterial community composition of sand and seawater. The correlations of environmental parameters and Bray–Curtis dissimilarity scores were assessed with the *envfit* function from ‘vegan’ (Oksanen et al. 2022). Partial Mantel tests, using Spearman's correlation co‐efficient, were then conducted to assess the relationship between bacterial community dissimilarity and temporal distance while controlling for geographic distance, and vice versa.

Bacterial species richness (observed ASVs, S_obs_) and bacterial species diversity (Inverse Simpson's index) of sand and seawater were calculated using the *estimate_richness* function of the ‘phyloseq’ package (McMurdie and Holmes 2013). Analysis of variance was used to assess differences in species diversity and richness between seawater/sand, date, and beach, followed by Tukey's HSD post hoc analyses as necessary. Pearson's correlation tests were used to examine relationships between bacterial species richness and diversity in sand and seawater samples. The influence of environmental parameters on richness and diversity was assessed with Pearson's correlation tests.

Relative abundances of dominant bacterial taxa (those accounting for > 1.0% of all 16S rRNA gene sequences) were compared between date and beach for sand and seawater using multivariate analysis of variance (MANOVA). The ‘core’ microbiome of sand or seawater was defined as the 10 most frequently detected ASVs above a per‐sample abundance threshold of 1% (i.e., > 1% of sequences within a given sample) using the ‘microbiome’ package (Lahti et al. 2017). Pearson's correlations were used to determine relationships between environmental variables and the relative abundance of dominant bacterial phyla and the relative abundance of ‘core’ ASVs for sand and seawater. Highly abundant ASVs (≥ 1.0% of sequences for seawater or > 0.45% for sand), that were not consistently found in all samples (i.e., not a part of the ‘core’ microbiome), were also included in these analyses.

Two‐way ANOVA was used to test for differences in seawater hydrolytic enzyme activity between sampling dates and beaches, and for differences in hydrolytic and oxidative enzyme activity for sand. Pearson's correlations were used to assess relationships between enzyme activity and environmental parameters, bacterial richness and diversity, relative abundances of dominant phyla, and ‘core’ and dominant ASVs to identify factors influencing bacterial community structure and function, with seawater and sand analysed separately. *Envfit* analyses were used to determine the association of enzyme activity in seawater and sand to respective bacterial communities.

## 3 Results

### 3.1 Variation in Environmental Parameters

**FIGURE 2:**
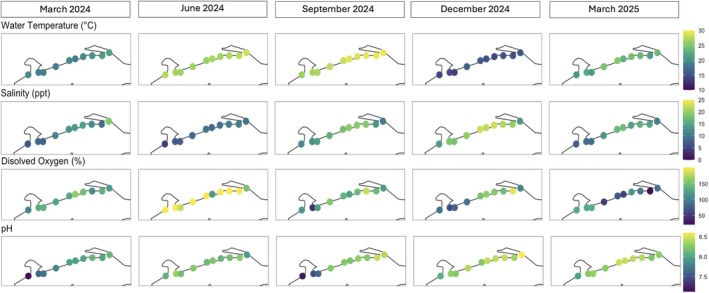
Environmental parameters (water temperature, °C; salinity, ppt; dissolved oxygen, %; pH) of seawater measured from 10 beaches along 53.1 km of the Mississippi coast, USA, once every 3 months from March 2024 to March 2025. Each panel depicts the unit of measurement for each parameter with colours indicating a range specific to that parameter.

Mean water temperature ranged from 14.8°C in December 2024 to 28.2°C in September 2024, with an overall mean of 22.5°C over the course of the study (Figure 2). Water temperature was routinely lowest at beach 6 (mean 21.3°C) and highest at beach 14 (mean 23.6°C). Salinity was lowest in June 2024 (mean 9.6 ppt) and highest in December 2024 (mean 19.3 ppt), with an overall mean of 13.5 ppt. Beach 4 had the lowest mean salinity at 8.8 ppt and beach 10 had the highest mean salinity (16.3 ppt). Seawater was highly saturated with dissolved oxygen (DO) which averaged 131.3% saturation (% S) overall, with the lowest mean in March 2025 (89.4% S) and the highest in June 2024 (178.8% S). Dissolved oxygen was lowest at beach 5 (mean 113.7% S) and highest at beach 11 (mean 153.1% S). Mean pH over the course of the study was 8.14, ranging from 7.82 in March 2024 to 8.35 in December 2024, and was lowest at beach 4 (mean 7.69) and highest at beach 12 (mean 8.27).

Temperature, salinity, and pH differed significantly between sampling dates (ANOVA; *F* = 9.21–350.1, *p* < 0.001) and between beaches (*F* = 3.14–6.09, *p* < 0.01–0.001) but DO did not (*p* > 0.05; Figure 2). After controlling for geographic distance, temporal distance (i.e., days between sampling dates) was positively correlated with differences in temperature (Mantel correlations; Spearman's correlation coefficient (*r*) = 0.12, *p* < 0.001), salinity (*r* = 0.12, *p* < 0.001), DO (*r* = 0.15, *p* < 0.001), and pH (*r* = 0.20, *p* < 0.001). When controlling for time, geographic distance between beaches was not correlated with temperature or DO (*p* > 0.05) but was significantly correlated with differences in salinity (*r* = 0.0916, *p* < 0.001) and pH (*r* = 0.24, *p* < 0.001).

### 3.2 Overall Patterns in the Bacterial Communities of Sand and Seawater

After removal of singletons, three samples each of sand and seawater were removed from the dataset because of low read counts (< 5000 sequences), leaving a total of 294 samples for analysis with a mean (± standard error) of 108,564 ± 10,255 sequence reads in sand samples and 167,221 ± 13,756 in seawater, and a median of 42,885 and 122,008 sequences, respectively. A total of 138,450 ASVs were in the initial dataset, with 21,016 ASVs used for downstream analysis after rarefaction to 5406 sequences per sample. Good's coverage scores for this rarefied dataset averaged 93.4% ± 0.002%.

**FIGURE 3:**
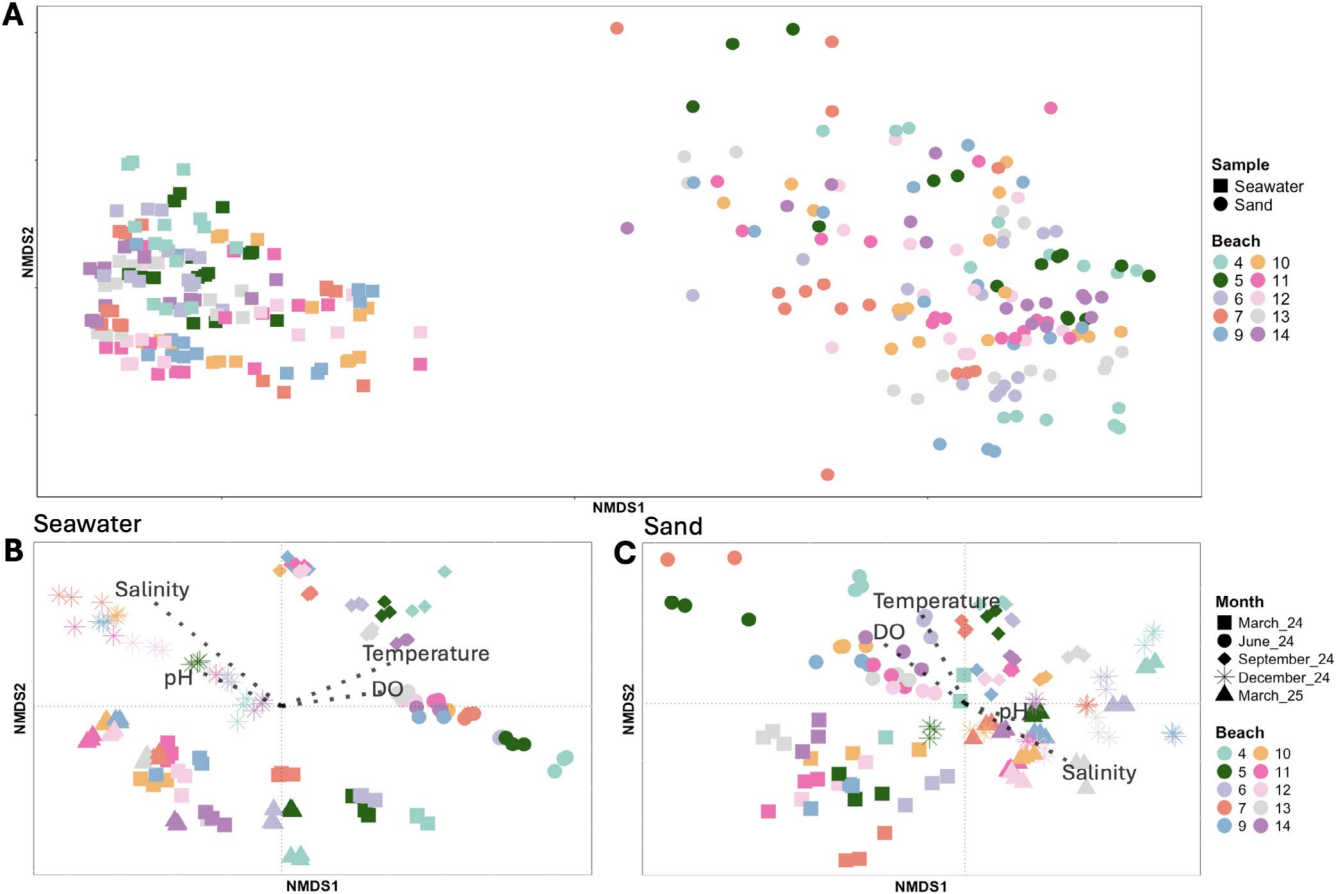
NMDS ordinations based on Bray–Curtis dissimilarity of bacterial communities from seawater and sand samples collected at 10 beaches along 53.1 km of the Mississippi coast, USA. Beach numbers correspond to designations used by the Mississippi Department of Environmental Quality. Samples were collected every 3 months from March 2024 to March 2025. Panels show a comparison of seawater and sand bacterial communities where sample type is indicated by shape and colour denotes the beach of collection (A), and separate spatiotemporal distribution of seawater (B) and sand (C) bacterial communities, with shape representing the month of collection and colour indicating the beach. Environmental vectors for temperature, salinity, dissolved oxygen (DO), and pH were fit to ordinations with the *envfit* function corresponding to bacterial community composition of seawater (B) and sand (C). Length and direction of vector arrows are proportionate to their effect size and association to the samples, respectively.

Bacterial community composition was significantly different between seawater and sand (PERMANOVA; *F* = 468.5, *R* ^2^ = 0.240, *p* < 0.001), by sample date (*F* = 82.9, *R* ^2^ = 0.167, *p* < 0.001), and by beach (*F* = 17.6, *R* ^2^ = 0.08, *p* < 0.001; Figure 3A). All of these factors combined to influence bacterial community composition and there were significant interaction effects for seawater/sand‐date (*F* = 58.3, *R* ^2^ = 0.117), seawater/sand‐beach (*F* = 14.4, *R* ^2^ = 0.065), date‐beach (*F* = 6.94, *R* ^2^ = 0.126), and seawater/sand‐date‐beach (*F* = 6.26, *R* ^2^ = 0.113; *p* < 0.001 for all). There was significant systematic dispersion detected between seawater and sand (permutest ANOVA; *p* < 0.001) but not based on sample date or beach (*p* > 0.05).

When analysing only seawater samples, bacterial community composition varied significantly by date (*F* = 170.3, *R* ^2^ = 0.522, *p* < 0.001) and beach sampled (*F* = 21.5, *R* ^2^ = 0.149, *p* < 0.001; Figure 3B), although dispersion across sample dates was significant (*p* < 0.001). After controlling for spatial distance, temporal distance was positively correlated with Bray–Curtis dissimilarity scores (Mantel correlations; Spearman's *r* = 0.45, *p* < 0.001). Distance between beaches retained a weaker but significant effect on seawater bacterial community structure when temporal variation was controlled (*r* = 0.14, *p* < 0.001). *Envfit* analysis indicated that variation in seawater bacterial community composition was best explained by salinity (*R* ^2^ = 0.85, *p* < 0.001), followed by temperature (*R* ^2^ = 0.39, *p* < 0.001), DO (*R* ^2^ = 0.26, *p* < 0.001), and pH (*R* ^2^ = 0.19, *p* < 0.001).

Bacterial community composition in sand differed significantly by date (*F* = 39.9, *R* ^2^ = 0.264, *p* < 0.001) and beach (*F* = 14.8, *R* ^2^ = 0.221, *p* < 0.001; Figure 3C). Temporal distance remained a strong predictor of community differences after controlling for spatial distance (*r* = 0.57, *p* < 0.001), whereas spatial distance between samples explained a smaller but still significant portion of variation after controlling for time (*r* = 0.25, *p* < 0.001). Water temperature (*R* ^2^ = 0.35, *p* < 0.001) and salinity (*R* ^2^ = 0.30, *p* < 0.001) were the strongest environmental predictors of variation in the composition of the sand bacterial community, followed by DO (*R* ^2^ = 0.24, *p* < 0.001), with pH being significant but explaining little variation (*R* ^2^ = 0.07, *p* < 0.01).

### 3.3 Alpha Diversity of Sand and Seawater Bacterial Communities

**FIGURE 4:**
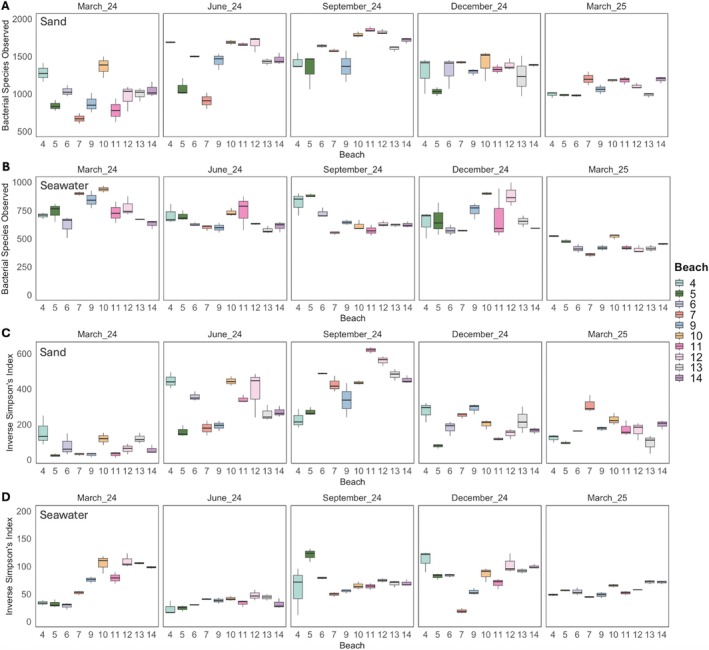
Bacterial species richness (S_obs_; A, B) and diversity (Inverse Simpson's index; C, D) of sand (A, C) and seawater (B, D) samples collected at 10 beaches along 53.1 km of the Mississippi coast, USA. Beach numbers correspond to designations used by the Mississippi Department of Environmental Quality. Samples were collected once every 3 months from March 2024 to March 2025, and each panel is one sample date. Each box shows the middle 50% of values (interquartile range), with the line inside the box representing the median. Whiskers indicate the range of values that fall within 1.5 times the interquartile range from the box limits. Note differences in y‐axis scales between sand and seawater for each metric.

Sand had significantly higher bacterial species richness (mean ± standard error (SE) S_obs_: 1308 ± 25; Figure 4A) than seawater (651 ± 13; ANOVA; *F* = 487.0, *p* < 0.001; Figure 4B) and significantly higher bacterial species diversity (mean ± SE inverse Simpson's index: 233.0 ± 12.7; Figure 4C) than seawater (62.9 ± 2.24; *F* = 186.6, *p* < 0.001; Figure 4D). Bacterial species richness showed significant interactions of date‐beach (*F* = 167.2, *p* < 0.001), seawater/sand‐beach (*F* = 30.8, *p* < 0.001), and seawater/sand‐date‐beach (*F* = 6.81, *p* < 0.001). Bacterial species diversity also showed significant interaction effects of seawater/sand‐date (*F* = 116.0, *p* < 0.001), seawater/sand‐beach (*F* = 11.3, *p* < 0.001), and seawater/sand‐date‐beach (*F* = 12.6, *p* < 0.001). Sand bacterial communities had significantly higher species richness than those in seawater on all sample dates (TukeyHSD; *p*‐adj < 0.001) and higher species diversity (*p*‐adj < 0.001) on all dates other than March 2024 (*p*‐adj > 0.05). The same pattern was true for each beach individually with greater species richness and diversity in sand than seawater at all 10 beaches (*p*‐adj < 0.001–0.05). Bacterial species diversity in sand and seawater collected on the same date and from the same beach was weakly negatively correlated (*r* = −0.18, *p* < 0.05), but bacterial species richness in the two habitats was not (*p* > 0.05).

In sand, observed bacterial species richness and diversity differed by date (*F* = 169.4, *p* < 0.001 and *F* = 310.1, *p* < 0.001, respectively) and beach (*F* = 18.7, *p* < 0.001 and *F* = 16.0, *p* < 0.001), with a significant date by beach interaction (*F* = 5.57, *p* < 0.001 and *F* = 11.4, *p* < 0.001). Sand species richness was highest in September 2024 (1621 ± 38) and lowest in March 2024 (986 ± 41). All pairwise comparisons of observed species richness between dates were significant (*p*‐adj < 0.001–0.05), except for between March 2024 and March 2025 (*p*‐adj > 0.05). In terms of spatial patterns, the sand bacterial community at beach 10 had significantly higher observed species richness (1486 ± 64) than that at beach 5 (1053 ± 50; *p*‐adj < 0.01), but no other pairwise comparisons between beaches were significant. As with species richness, species diversity of the sand bacterial community was highest in September 2024 (mean + SE inverse Simpson's index: 429.6 ± 23.7) and lowest in March 2024 (69.8 ± 10.1), and all pairwise comparisons of sand species diversity between dates were significant (*p*‐adj < 0.001), except for between December 2024 and March 2025 (*p*‐adj > 0.05). No pairwise comparisons of sand bacterial diversity between individual beaches were significant (*p*‐adj > 0.05). Sand species richness was positively correlated with water temperature (*r* = 0.49, *p* < 0.001) and DO (*r* = 0.29, *p* < 0.001), as was species diversity (temperature: *r* = 0.63, *p* < 0.001 and DO: *r* = 0.22, *p* < 0.01). Neither sand community richness nor diversity were significantly correlated with pH or salinity (*p* > 0.05 for all).

As with sand, observed species richness and diversity of the seawater bacterial community differed by date (richness *F* = 100.7, *p* < 0.001; diversity *F* = 84.1, *p* < 0.001) and beach (richness *F* = 12.6, *p* < 0.001; diversity *F* = 19.1, *p* < 0.001), with a significant date by beach interaction (richness *F* = 5.21, *p* < 0.001; diversity *F* = 11.8, *p* < 0.001). The highest observed species richness in the seawater bacterial community was in March 2024 (761 ± 23), which was significantly higher than seawater species richness in June 2024 (653 ± 15), September 2024 (667 ± 20), and March 2025 (438 ± 9; *p*‐adj < 0.01 for all). The lowest seawater bacterial species richness was in March 2025, which was significantly lower than that in June 2024 and December 2024 (*p*‐adj < 0.001 for both). While observed bacterial species richness in seawater differed between the 10 beaches overall, no individual pairwise comparisons between beaches in terms of species richness were significant (*p*‐adj > 0.05). Species diversity in the seawater bacterial community was significantly lower in June 2024 (35.4 ± 1.84) than the other sampling dates (*p*‐adj < 0.001), and species diversity in March 2025 (56.9 ± 1.75) was lower than that in December 2024 (79.5 ± 5.18; *p*‐adj < 0.01). In terms of beaches, seawater at beach 7 had the lowest bacterial species diversity (41.2 ± 3.20), which was lower than that at beach 10 (72.4 ± 6.16), 12 (78.3 ± 6.90), 13 (72.9 ± 6.00), and 14 (73.9 ± 6.70; *p*‐adj < 0.05 for all). Seawater observed bacterial species richness was significantly negatively correlated with water temperature (*r* = −0.21, *p* < 0.05), DO (*r* = −0.42, *p* < 0.05), and pH (*r* = −0.29, *p* < 0.05), but not with salinity (*p* > 0.05). Seawater bacterial species diversity was negatively correlated with temperature (*r* = −0.36, *p* < 0.001) and DO (*r* = −0.32, *p* < 0.001), and positively correlated with salinity (*r* = 0.40, *p* < 0.001), but not correlated with pH (*p* > 0.05).

### 3.4 Taxonomic Composition of Sand and Seawater Bacterial Communities

**FIGURE 5:**
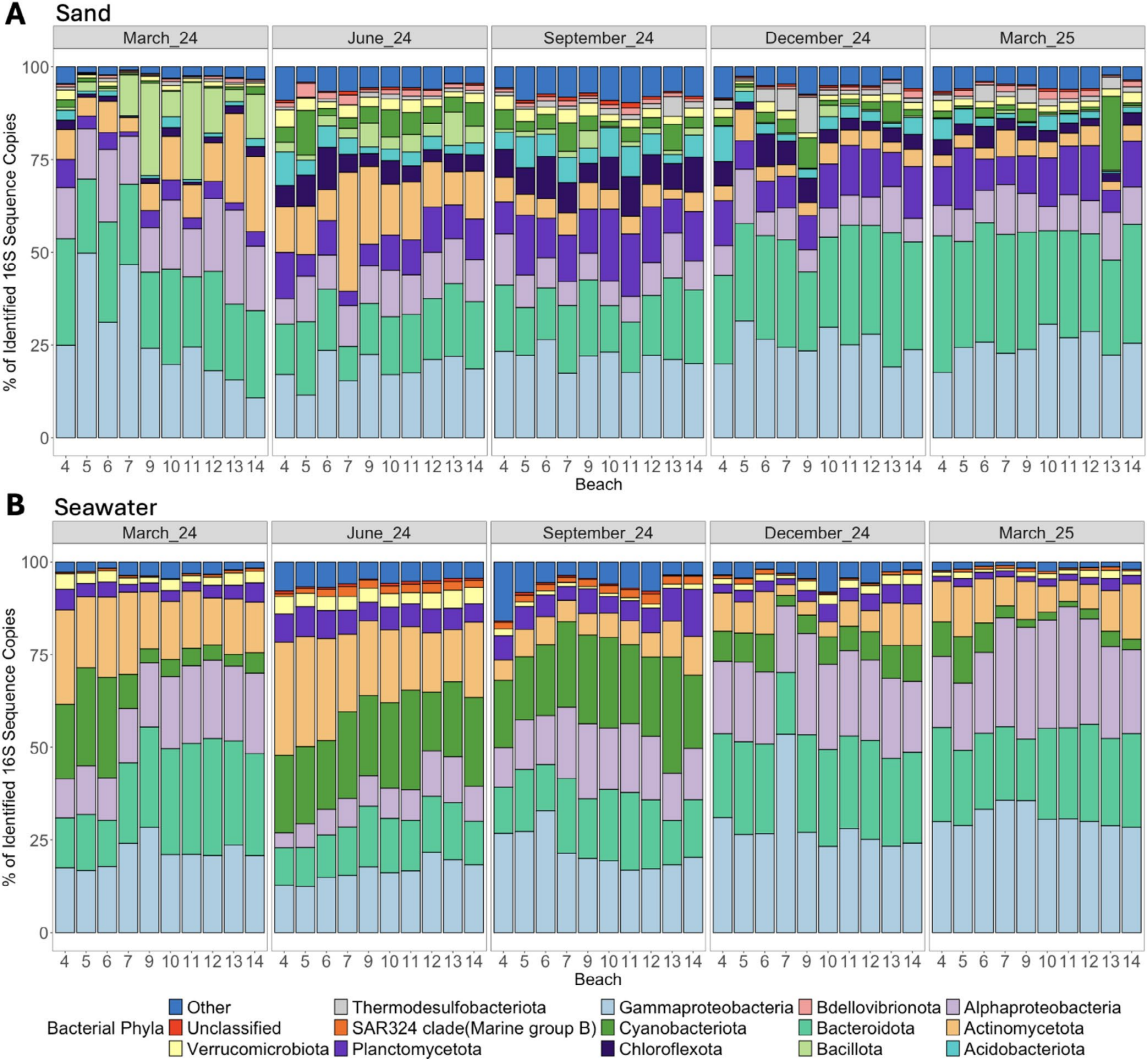
Proportions of dominant bacterial phyla in the bacterial community of sand (A) and seawater (B) samples collected at 10 beaches along 53.1 km of the Mississippi coast, USA. Beach numbers correspond to designations used by the Mississippi Department of Environmental Quality. Samples were collected once every 3 months from March 2024 to March 2025, with each panel being one sample date. Each bar represents the mean relative abundance of phyla from three samples of sand or seawater at the corresponding collection date and beach. ‘Unclassified’ represents bacterial sequences that were not identified to a phylum, whereas ‘Other’ represents bacterial sequences identified to a phylum that did not classify within the most abundant phyla (defined as accounting for > 1.0% of all bacterial sequences identified in sand or seawater).

Bacterial communities were primarily composed of Gammaproteobacteria (23.3% of sequences recovered from sand; 24.2% from seawater) and Bacteroidota (22.9% sand and 19.8% seawater), with other abundant phyla being Alphaproteobacteria (11.0% and 17.6% in sand and seawater, respectively), Actinomycetota (7.86% and 12.7%), Planctomycetota (9.69% and 4.39%), Cyanobacteriota (3.30% and 13.3%), and Verrucomicrobiota (2.15% and 2.26%) (Figure 5). Fewer than 1% of sequences could not be classified to a phylum (i.e., “Unclassified”). Sand bacterial communities also contained high proportions of sequences classified as Chloroflexota (4.69% of the community), Acidobacteriota (3.47%), Bacillota (3.32%), Thermodesulfobacteriota (1.64%), and Bdellovibrionota (1.06%; Figure 5A), whereas seawater bacterial communities contained a noticeable amount of the SAR324 clade (Marine group B; 1.20% of sequences; Figure 5B). The proportions of phyla in sand and seawater bacterial communities differed significantly by sampling date (MANOVA; *F* = 6.87–103.4, *p* < 0.001). In the sand community, the proportions of all major phyla except Bacteroidota and Actinomycetota also differed between beaches (*F* = 2.17–6.18, *p* < 0.001–0.05). For seawater, only the proportions of Bacteroidota, Alphaproteobacteria, and Actinomycetota differed by beach (*F* = 2.24–3.60, *p* < 0.001–0.05).

Several of the dominant bacterial phyla in sand showed significant correlations with environmental parameters. Temperature was positively correlated with proportions of Actinomycetota (*r* = 0.24, *p* < 0.01), Planctomycetota (*r* = 0.28, *p* < 0.001), Cyanobacteriota (*r* = 0.23, *p* < 0.01), Verrucomicrobiota (*r* = 0.45, *p* < 0.001), Chloroflexota (*r* = 0.44, *p* < 0.001), Acidobacteriota (*r* = 0.35, *p* < 0.001), and Bdellovibrionota (*r* = 0.46, *p* < 0.001) in the sand bacterial community, but negatively correlated with proportions of Gammaproteobacteria (*r* = −0.34, *p* < 0.001), Bacteroidota (*r* = −0.58, *p* < 0.001), and Thermodesulfobacteriota (*r* = −0.21, *p* < 0.05). Salinity was positively correlated with proportions of Bacteroidota (*r* = 0.20, *p* < 0.01), Planctomycetota (*r* = 0.31, *p* < 0.001), and Thermodesulfobacteriota (*r* = 0.41, *p* < 0.001) in sand, but negatively correlated with proportions of Alphaproteobacteria (*r* = −0.33, *p* < 0.001), Actinomycetota (*r* = −0.42, *p* < 0.001), Verrucomicrobiota (*r* = −0.24, *p* < 0.01), and Bdellovibrionota (*r* = −0.22, *p* < 0.01). DO was positively correlated with proportions of Actinomycetota (*r* = 0.42, *p* < 0.001), Bacillota (*r* = 0.17, *p* < 0.05), and Bdellovibrionota (*r* = 0.22, *p* < 0.01), and negatively correlated with proportions of Gammaproteobacteria (*r* = −0.25, *p* < 0.01) and Bacteroidota (*r* = −0.35, *p* < 0.001), while pH was positively correlated with proportions of Bacteroidota (*r* = 0.24, *p* < 0.01), Planctomycetota (*r* = 0.30, *p* < 0.001), Thermodesulfobacteriota (*r* = 0.30, *p* < 0.001), and Bdellovibrionota (*r* = 0.27, *p* < 0.001) and negatively correlated with the proportion of Alphaproteobacteria (*r* = −0.35, *p* < 0.001).

In the seawater community, temperature was positively correlated with proportions of Planctomycetota (*r* = 0.53, *p* < 0.001), Cyanobacteriota (*r* = 0.57, *p* < 0.001), and SAR324 clade Marine group B (*r* = 0.70, *p* < 0.001), but negatively correlated with proportions of Gammaproteobacteria (*r* = −0.37, *p* < 0.001), Bacteroidota (*r* = −0.58, *p* < 0.001), and Alphaproteobacteria (*r* = −0.38, *p* < 0.001). Salinity was positively correlated with proportions of Gammaproteobacteria (*r* = 0.36, *p* < 0.001), Bacteroidota (*r* = 0.49, *p* < 0.001), and Alphaproteobacteria (*r* = 0.61, *p* < 0.001), but negatively correlated with proportions of Actinomycetota (*r* = −0.76, *p* < 0.001), Planctomycetota (*r* = −0.24, *p* < 0.001), Cyanobacteriota (*r* = −0.37, *p* < 0.001), and Verrucomicrobiota (*r* = −0.45, *p* < 0.001). DO was positively correlated with proportions of Actinomycetota (*r* = 0.40, *p* < 0.001), Planctomycetota (*r* = 0.42, *p* < 0.001), Cyanobacteriota (*r* = 0.47, *p* < 0.001), Verrucomicrobiota (*r* = 0.51, *p* < 0.001), and SAR324 clade Marine group B (*r* = 0.34, *p* < 0.001) in seawater, but negatively correlated with proportions of Gammaproteobacteria (*r* = −0.58, *p* < 0.001), Bacteroidota (*r* = −0.30, *p* < 0.001), and Alphaproteobacteria (*r* = −0.53, *p* < 0.001), while pH showed positive correlations with proportions of Bacteroidota (*r* = 0.28, *p* < 0.001) and Alphaproteobacteria (*r* = 0.43, *p* < 0.001), but negative correlations with proportions of Actinomycetota (*r* = −0.30, *p* < 0.001), Cyanobacteriota (*r* = −0.28, *p* < 0.001), and Verrucomicrobiota (*r* = −0.18, *p* < 0.05).

### 3.5 Patterns in the ‘Core’ Bacterial Microbiome of Sand and Seawater

**FIGURE 6:**
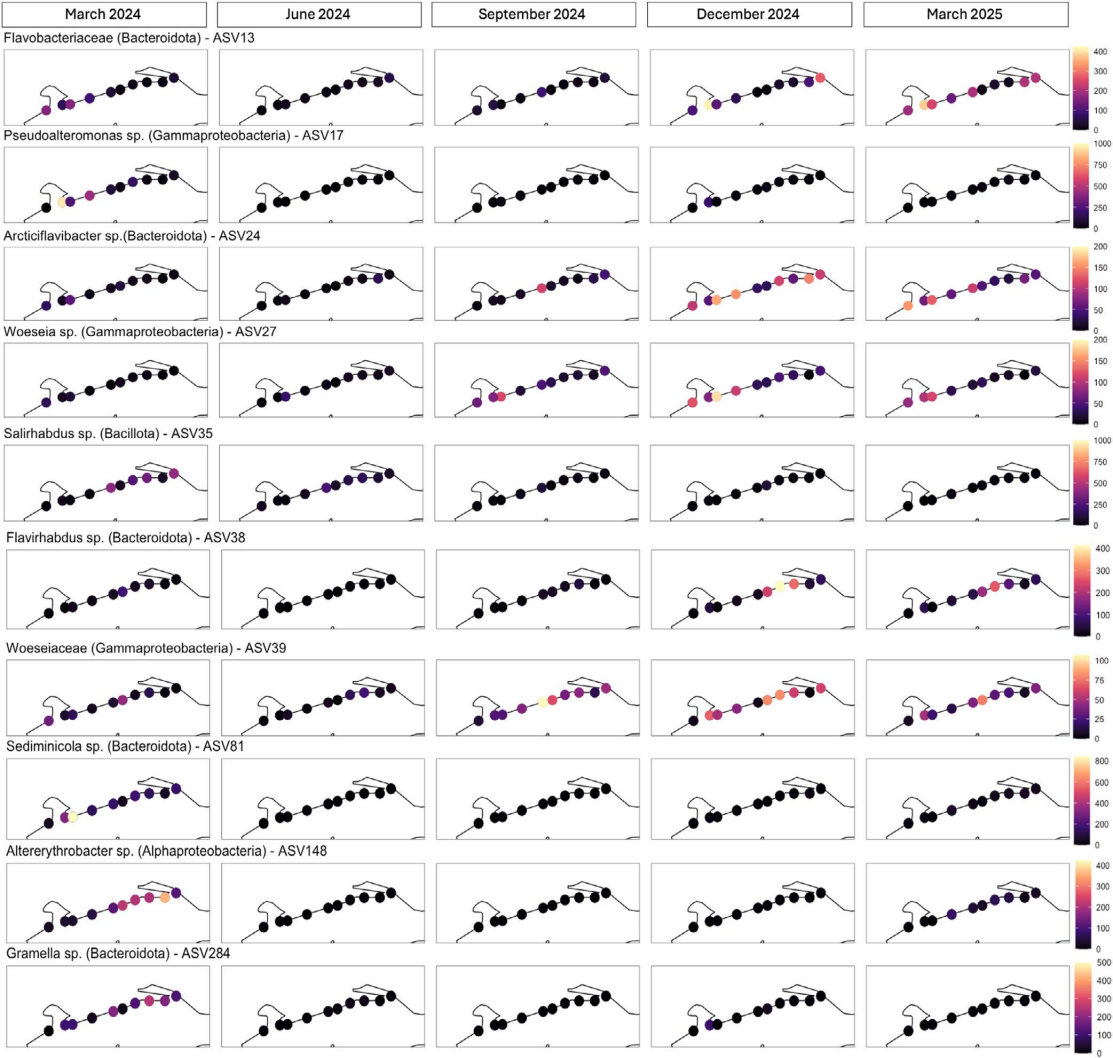
Spatial and temporal distribution of bacterial ASVs in sand collected at 10 beaches along 53.1 km of the Mississippi coast, USA, once every 3 months from March 2024 to March 2025. Each panel depicts the abundance (number of sequences out of 5406) of a single ASV at each beach on that sample date, with colours indicating an abundance range specific to each ASV.

At a finer taxonomic resolution, the members of the ‘core’ microbiome (defined here as the 10 most frequently detected ASVs that each comprised > 1% of sequences in each sample of that habitat type) in sand were ASV 13 (Flavobacteriaceae; Bacteroidota), ASV 17 (*Pseudoalteromonas* sp.; Gammaproteobacteria), ASV 24 (*Arcticiflavibacter* sp.; Bacteroidota), ASV 27 (*Woeseia* sp.; Gammaproteobacteria), ASV 35 (*Salirhabdus* sp.; Bacillota), ASV 38 (*Flavirhabdus* sp.; Bacteroidota), ASV 39 (Woeseiaceae; Gammaproteobacteria), ASV 81 (*Sediminicola* sp.; Bacteroidota), ASV 148 (*Altererythrobacter* sp.; Alphaproteobacteria), and ASV 284 (*Gramella* sp.; Bacteroidota). Four additional ASVs, ASV 106 (*Psychrobacter*; Gammaproteobacteria), ASV 160 (*Gramella*; Bacteroidota), ASV 204 (*Planococcus*; Bacillota), and ASV 139 (*Anaerobacillus*; Bacillota), were abundant in sand (each > 0.45% of all sequences detected in sand). The relative abundance of these ‘core’ and abundant ASVs in the sand bacterial community differed by date (ANOVA; *F* = 34.8–325.0, *p* < 0.001) and beach (*F* = 3.10–168.2, *p* < 0.001; Figure 6) and showed some correlation with environmental parameters (Table 1). Temperature was positively correlated with the percentage of ASV 139 and negatively correlated with ASV 24, while salinity was positively correlated with ASV 38 and ASV 39 and DO was negatively correlated with ASV 13. No strong (*r* > 0.40) correlations were observed between pH and the most abundant ASVs of sand. Several of these ASVs were associated with specific sampling dates and beach locations (*envfit* analysis of Bray–Curtis dissimilarity scores; *p* < 0.001 for all). ASV 13 (*R* ^2^ = 0.13) and ASV 38 (*R* ^2^ = 0.15) were primarily associated with sand bacterial communities in March 2025, with ASV 38 particularly associated with beaches 10, 11, and 12. ASV 24 (*R* ^2^ = 0.46) and ASV 39 (*R* ^2^ = 0.16) were associated with samples from December 2024 and March 2025 but showed no association to specific beaches. ASV 17 (*R* ^2^ = 0.28), ASV 35 (*R* ^2^ = 0.30), ASV 81 (*R* ^2^ = 0.27), ASV 148 (*R* ^2^ = 0.39), ASV 284 (*R* ^2^ = 0.36), ASV 106 (*R* ^2^ = 0.30), ASV 160 (*R* ^2^ = 0.29), and ASV 204 (*R* ^2^ = 0.25) were all associated with March 2024, and also showed no association to the beach sampled. ASV 27 (*R* ^2^ = 0.38) was specifically associated with September 2024 samples from beach 13 and ASV 139 (*R* ^2^ = 0.28) with June 2024 samples overall.

**TABLE 1:** Major ASVs in bacterial communities in seawater and sand collected from 10 beaches along 53.1 km of the Mississippi coast in March 2024, June 2024, September 2024, December 2024, and March 2025.

Sand						
ASV#	Taxonomy	Relative abundance	DO	pH	Salinity	Temperature
13	Flavobacteriaceae	1.45%	−0.42	—	—	−0.33
35	*Salirhabdus*	1.32%	—	—	—	—
17	*Pseudoalteromonas*	0.91%	—	−0.32	—	−0.25
38	*Flavirhabdus*	0.80%	—	0.39	0.53	−0.35
24	*Arcticiflavibacter*	0.79%	−0.33	0.33	0.24	−0.50
106	*Psychrobacter*	0.72%	—	—	—	−0.16
81	*Sediminicola*	0.70%	—	−0.25	−0.23	0.22
148	*Altererythrobacter*	0.63%	—	—	—	−0.18
27	*Woeseia*	0.58%	−0.38	—	—	−0.28
160	*Gramella*	0.55%	—	—	—	—
204	*Planococcus*	0.55%	—	—	—	—
39	Woeseiaceae	0.51%	−0.30	0.36	0.55	—
139	*Anaerobacillus*	0.47%	0.36	—	−0.28	0.43
284	*Gramella*	0.44%	—	—	—	—

Seawater						
ASV#	Taxonomy	Relative abundance	DO	pH	Salinity	Temperature
1	*Cyanobium PCC‐6307*	3.75%	0.36	−0.47	−0.65	0.25
2	Sporichthyaceae – *hgcl clade*	2.85%	0.54	—	−0.68	0.37
3	*Cyanobium PCC‐6307*	2.50%	0.39	—	—	0.62
7	Rhodobacteraceae	2.40%	—	0.47	0.85	−0.20
4	*Candidatus Actinomarina*	2.39%	0.44	−0.34	−0.58	0.18
5	SAR 86 clade	2.28%	−0.44	0.4	0.47	−0.45
9	*Candidatus Aquiluna*	1.70%	−0.45	—	—	−0.23
11	Methylophilaceae	1.63%	−0.37	—	−0.38	—
10	*Candidatus Actinomarina*	1.52%	−0.24	—	0.53	−0.28
6	SAR 86 clade	1.50%	0.42	—	—	0.60
8	AEGEAN‐169 marine group	1.48%	−0.18	0.3	−0.17	—
14	*Litorimicrobium*	1.28%	−0.31	—	−0.19	−0.31
16	*Synechococcus CC9902*	1.00%	−0.17	—	0.37	0.75

*Note:* Within each sample type (seawater or sand), ASVs are ordered by relative abundance (highest to lowest), with the finest assigned taxonomic resolution shown. Major ASVs were determined to be those accounting for ≥ 1.0% of sequences recovered from seawater or ≥ 0.4% of sequences from sand. Pearson's correlation coefficients between ASV abundance and environmental variables (dissolved oxygen (DO), pH, salinity, and temperature) are reported where significant (*p* < 0.05 to *p* < 0.001); dashes indicate no significant correlations (*p* > 0.05).

**FIGURE 7:**
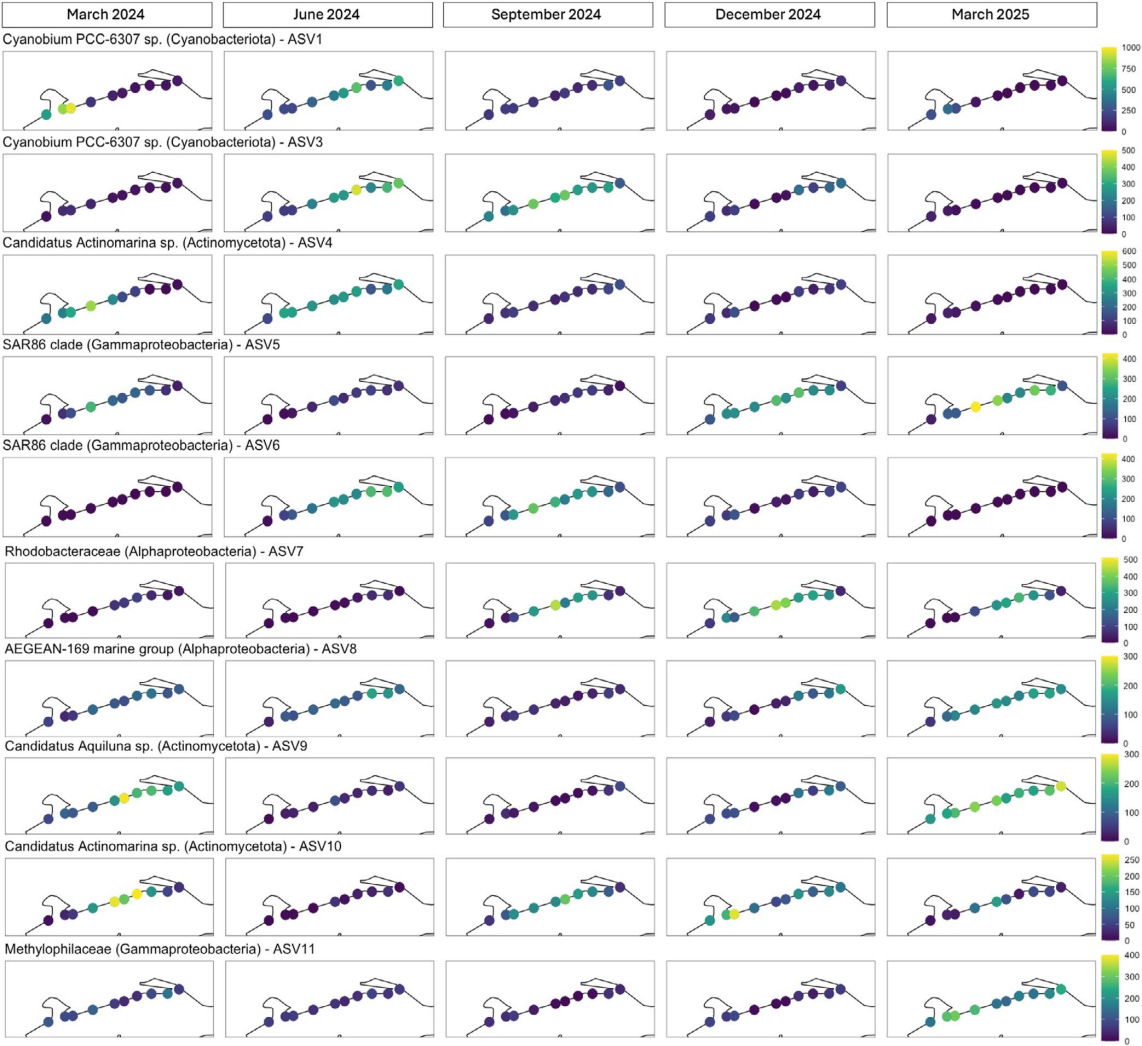
Spatial and temporal distribution of bacterial ASVs in seawater collected at 10 beaches along 53.1 km of the Mississippi coast, USA, once every 3 months from March 2024 to March 2025. Each panel depicts the abundance (number of sequences out of 5406) of a single ASV at each beach on that sample date, with colours indicating an abundance range specific to each ASV.

The ‘core’ ASVs in seawater were ASV 1 and ASV 3 (both classified as *Cyanobium PCC‐6307* sp.; Cyanobacteriota), ASV 4 and ASV 10 (both *Candidatus Actinomarina* sp.; Actinomycetota), ASV 5 and ASV 6 (both SAR86 clade; Gammaproteobacteria), ASV 7 (Rhodobacteraceae; Alphaproteobacteria), ASV 8 (AEGEAN‐169 marine group; Alphaproteobacteria), ASV 9 (*Candidatus Aquiluna* sp.; Actinomycetota), and ASV 11 (Methylophilaceae; Gammaproteobacteria). Three additional ASVs each accounted for > 1.0% of the total seawater sequences but were not detected that frequently in all samples: ASV 2 (*hgcI clade*; Actinomycetota), ASV 14 (*Litorimicrobium*; Alphaproteobacteria), and ASV 16 (*Synechococcus* CC9902; Cyanobacteriota). The relative abundance of these ASVs in seawater differed by date (*F* = 134.5–1046.7, *p* < 0.001) and beach (*F* = 6.8–45.6, *p* < 0.001; Figure 7). Percentages of these ASVs in the seawater community were generally more correlated with environmental parameters than ASVs in the sand bacterial community, and several showed strong correlations with temperature or salinity (Table 1). Temperature was positively correlated with the percentage of ASV 3, ASV 6, and ASV 2, and negatively with ASV 5 and ASV 14. Salinity was positively correlated with the percentage of ASV 7, ASV 16, ASV 10, and ASV 5, but negatively correlated with ASV 2, ASV 1, ASV 4, and ASV 14. DO was positively correlated with ASV 2 and ASV 6 and negatively correlated with ASV 9, ASV 11, and ASV 14. pH was positively correlated with ASV 7 and ASV 5 while negatively correlated with the percentage of ASV 1. *Envfit* analysis of Bray–Curtis dissimilarity scores also revealed temporal and spatial patterns in the distribution of these ASVs. ASV 1 (*R* ^2^ = 0.58), ASV 2 (*R* ^2^ = 0.68), ASV 4 (*R* ^2^ = 0.52) were strongly associated with samples collected in March and June 2024, particularly from beaches 4, 5, and 6 (*p* < 0.001 for all). ASV 5 (*R* ^2^ = 0.59), ASV 8 (*R* ^2^ = 0.24), ASV 9 (*R* ^2^ = 0.73), ASV 11 (*R* ^2^ = 0.55), and ASV 14 (*R* ^2^ = 0.49) were associated with seawater collected in March 2024 and March 2025 (*p* < 0.001 for all). Among these, ASV 5 was primarily associated with the centrally located sites (beaches 9–12), while ASVs 8, 9, and 11 were associated with the easternmost beaches (sites 13 and 14). ASV 3 (*R* ^2^ = 0.69) and ASV 6 (*R* ^2^ = 0.61) were associated with seawater communities sampled in September 2024, whereas ASV 7 (*R* ^2^ = 0.77), ASV 10 (*R* ^2^ = 0.30), and ASV 16 (*R* ^2^ = 0.53; *p* < 0.001 for all) were associated with samples from December 2024.

### 3.6 Relationships of Bacterial Community Composition to Enzyme Activity

**FIGURE 8:**
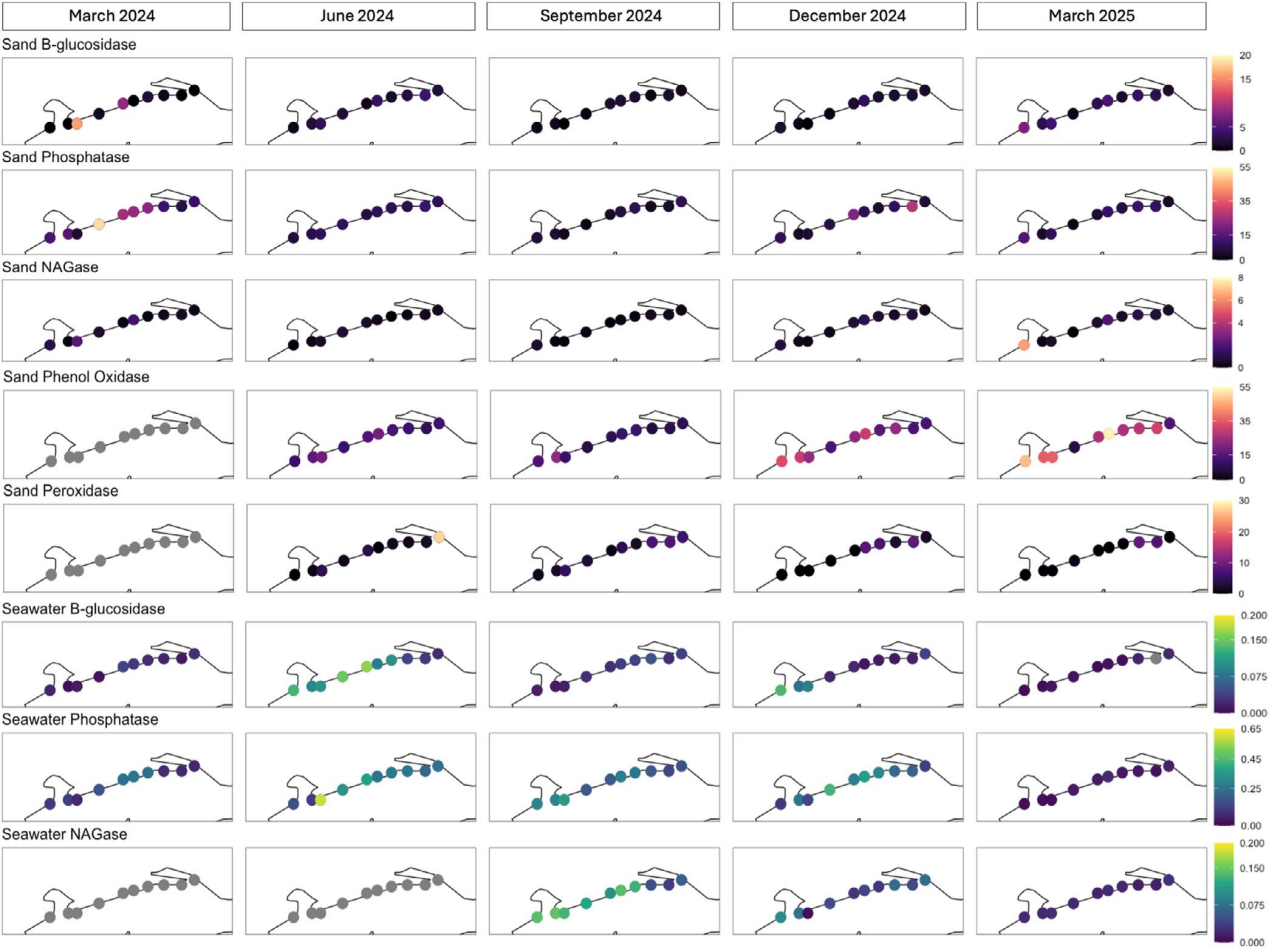
Extracellular enzyme activity in sand (nmol h^−1^ g^−1^) and seawater (nmol h^−1^ L^−1^) collected from 10 beaches along 53.1 km of the Mississippi coast, USA, once every 3 months from March 2024 to March 2025. Each panel depicts the activity of a specific enzyme with colours indicating a range of activity specific to that enzyme.

In sand, activities of β‐glucosidase, NAGase, and phenol oxidase varied significantly by date (*F* = 3.04–97.5, *p* < 0.001–0.05) and beach (*F* = 3.16–19.0, *p* < 0.001–0.05), but activities of phosphatase and peroxidase did not (*p* > 0.05; Figure 8). There was a significant date‐beach interaction for the activities of all enzymes (*F* = 1.57–3.12, *p* < 0.001–0.05). Activities of the three hydrolytic enzymes were positively correlated with each other (β‐glucosidase—phosphatase *r* = 0.18, *p* < 0.05; β‐glucosidase—NAGase *r* = 0.48, *p* < 0.001; phosphatase—NAGase *r* = 0.33, *p* < 0.001), whereas activities of the two oxidative enzymes (phenol oxidase, peroxidase) were negatively correlated (*r* = −0.24, *p* < 0.01). In terms of environmental factors, temperature was positively correlated with sand peroxidase activity (*r* = 0.23, *p* < 0.05) but negatively correlated with activities of phosphatase (*r* = −0.31, *p* < 0.01), NAGase (*r* = −0.26, *p* < 0.01), and phenol oxidase (*r* = −0.52, *p* < 0.001). Salinity was weakly negatively correlated with sand β‐glucosidase activity (*r* = −0.19, *p* < 0.05) but showed no correlations to other enzymes (*p* > 0.05), while DO was negatively correlated with phenol oxidase activity (*r* = −0.38, *p* < 0.001), and pH was negatively correlated with phosphatase activity (*r* = −0.23, *p* < 0.01). For community metrics, sand bacterial species richness was negatively correlated with the activities of β‐glucosidase (*r* = −0.21, *p* < 0.05), phosphatase (*r* = −0.37, *p* < 0.001), NAGase (*r* = −0.30, *p* < 0.001), and phenol oxidase (*r* = −0.62, *p* < 0.001), but positively with peroxidase (*r* = 0.23, *p* < 0.05). Sand bacterial diversity was negatively correlated with the activities of phosphatase (*r* = −0.37, *p* < 0.001), NAGase (*r* = −0.28, *p* < 0.001), and phenol oxidase (*r* = −0.59, *p* < 0.001).

Activities of β‐glucosidase, phosphatase, and NAGase in seawater also differed by date (*F* = 138.4–351.0, *p* < 0.001) and beach (*F* = 9.36–15.1, *p* < 0.001), with significant date‐beach interactions (*F* = 5.21–15.1, *p* < 0.001; Figure 8). Activities of β‐glucosidase and phosphatase were correlated with each other (*r* = 0.35, *p* < 0.001), and seawater phosphatase activity was also correlated with seawater NAGase activity (*r* = 0.20, *p* < 0.05). Water temperature was not significantly correlated with the activity of any enzyme in seawater (*p* > 0.05), while salinity was negatively correlated with the activity of β‐glucosidase (*r* = −0.25, *p* < 0.01) and positively correlated with the activity of NAGase (*r* = 0.42, *p* < 0.001). DO was positively correlated with the activities of β‐glucosidase (*r* = 0.35, *p* < 0.001) and phosphatase (*r* = 0.38, *p* < 0.001), and negatively correlated with NAGase activity (*r* = −0.31, *p* < 0.001), which was also negatively correlated with pH (*r* = −0.19, *p* < 0.05). In terms of community metrics, species richness of the seawater bacterial community was positively correlated with phosphatase activity (*r* = 0.48, *p* < 0.001), but not with the activities of β‐glucosidase or NAGase (*p* > 0.05), while species diversity was only correlated with the activity of NAGase (*r* = 0.41, *p* < 0.001).

In sand, β‐glucosidase activity was positively correlated with the proportion of Bacteroidota in the sand bacterial community (*r* = 0.19, *p* < 0.05) and negatively correlated with the proportion of Chloroflexota (*r* = −0.18, *p* < 0.05). Phosphatase activity was positively correlated with proportions of Gammaproteobacteria (*r* = 0.23, *p* < 0.01), Alphaproteobacteria (*r* = 0.24, *p* < 0.01), and Bacillota (*r* = 0.37, *p* < 0.001) and negatively correlated with Planctomycetota (*r* = −0.39, *p* < 0.001), Verrucomicrobiota (*r* = −0.32, *p* < 0.001), Chloroflexota (*r* = −0.33, *p* < 0.001), Acidobacteriota (*r* = −0.28, *p* < 0.001), and Bdellovibrionota (*r* = −0.33, *p* < 0.001). Sand NAGase activity was positively correlated with the proportion of Bacteroidota (*r* = 0.30, *p* < 0.001) and negatively correlated with proportions of Actinomycetota (*r* = −0.57, *p* < 0.001), Verrucomicrobiota (*r* = −0.56, *p* < 0.001), Chloroflexota (*r* = −0.25, *p* < 0.01), and Bdellovibrionota (*r* = −0.25, *p* < 0.01). Of the two oxidative enzymes assayed in sand, phenol oxidase activity was positively correlated with the proportions of Gammaproteobacteria (*r* = 0.43, *p* < 0.001) and Bacteroidota (*r* = 0.48, *p* < 0.001) in the sand bacterial community, and negatively correlated with Actinomycetota (*r* = −0.34, *p* < 0.001), Verrucomicrobiota (*r* = −0.38, *p* < 0.001), Chloroflexota (*r* = −0.34, *p* < 0.001), Bacillota (*r* = −0.34, *p* < 0.001), and Bdellovibrionota (*r* = −0.25, *p* < 0.01). The only significant correlation between sand peroxidase activity and bacterial phyla was with Planctomycetota (*r* = 0.23, *p* < 0.05). There were fewer strong correlations between sand enzyme activity and the relative abundance of specific ASVs. Sand phosphatase activity was positively correlated with the percentage of ASV 17 (*r* = 0.44, *p* < 0.001), ASV 81 (*r* = 0.48, *p* < 0.001), ASV 106 (*r* = 0.41, *p* < 0.001), and ASV 204 (*r* = 0.41, *p* < 0.001), while sand phenol oxidase activity was positively correlated with the percentage of ASV 13 and ASV 24 (both *r* = 0.41, *p* < 0.001).

Seawater also showed correlations between proportions of bacterial phyla in the community and the activities of β‐glucosidase, phosphatase, and NAGase. β‐glucosidase activity was positively correlated with proportions of Actinomycetota (*r* = 0.41, *p* < 0.001), Planctomycetota (*r* = 0.29, *p* < 0.001), Cyanobacteriota (*r* = 0.29, *p* < 0.001), Verrucomicrobiota (*r* = 0.37, *p* < 0.001), and SAR324 clade Marine group B (*r* = 0.38, *p* < 0.001), but showed negative correlations with the proportions of Gammaproteobacteria (*r* = −0.30, *p* < 0.001), Bacteroidota (*r* = −0.31, *p* < 0.001), and Alphaproteobacteria (*r* = −0.53, *p* < 0.001). Phosphatase activity in seawater was positively correlated with proportions of Planctomycetota (*r* = 0.41, *p* < 0.001), Cyanobacteriota (*r* = 0.29, *p* < 0.001), Verrucomicrobiota (*r* = 0.22, *p* < 0.01), and SAR324 clade Marine group B (*r* = 0.39, *p* < 0.001), and negatively correlated with proportions of Bacteroidota (*r* = −0.31, *p* < 0.001) and Alphaproteobacteria (*r* = −0.45, *p* < 0.001). Seawater NAGase activity was positively correlated with the proportion of Gammaproteobacteria (*r* = 0.20, *p* < 0.05) but was strongly negatively correlated with Actinomycetota (*r* = −0.57, *p* < 0.001) and Verrucomicrobiota (*r* = −0.56, *p* < 0.001). Several strong correlations existed between seawater enzyme activity and the relative abundance of individual ASVs. β‐glucosidase activity was positively correlated with the percentage of ASV 2 (*r* = 0.53, *p* < 0.001) and ASV 4 (*r* = 0.40, *p* < 0.001) but negatively correlated with ASV 9 (*r* = −0.40, *p* < 0.001). Phosphatase activity was positively correlated with the percentage of ASV 6 (*r* = 0.42, *p* < 0.001) and negatively correlated with ASV 9 (*r* = −0.62, *p* < 0.001), ASV 11 (*r* = −0.64, *p* < 0.001), and ASV 14 (*r* = −0.56, *p* < 0.001). Seawater NAGase activity was positively correlated with the percentage of ASV 10 (*r* = 0.44, *p* < 0.001) and ASV 16 (*r* = 0.55, *p* < 0.001) but negatively correlated with percentages of ASV 2 (*r* = −0.47, *p* < 0.001) and ASV 8 (*r* = −0.49, *p* < 0.001).

## 4 Discussion

Microbial communities in coastal systems are shaped by a variety of factors that vary temporally and spatially, yet the influence of these factors can differ across habitats (Fuhrman et al. 2015; Halliday et al. 2014). Here, we examined the temporal and spatial variability in bacterial communities in sand and seawater along a > 50 km stretch of coastline over a 12‐month period, with samples collected from 10 beaches every 3 months from March 2024 to March 2025. We found that the bacterial community structure of both sand and seawater varied more strongly with time than with space, consistent with previous analyses of temporal variation in marine microbial communities (Ferrera et al. 2024; Fuhrman et al. 2006; Krabberod et al., Krabberød et al. 2022; Wang et al. 2020; Ward et al. 2017). Sand and seawater bacterial communities clearly separated based on sampling date, with a potential annual or seasonal cyclic pattern seen in seawater, which showed similar bacterial communities in March 2024 and March 2025. This cyclic pattern was not seen in the sand bacterial community, and the two habitats differed in the apparent influence of environmental factors. Variation in the composition of the sand bacterial community most correlated with water temperature, whereas seawater bacterial communities were more closely correlated to patterns in salinity.

Sand consistently supported a richer and more diverse bacterial community than seawater, likely because of the higher diversity of niches available in sand (Gobet et al. 2012; Mills et al. 2008). As with community composition, the environmental drivers that may have shaped bacterial species diversity and richness differed between the two habitats with higher temperatures and dissolved oxygen (DO) correlating with higher richness and diversity in sand. These parameters correlated with lower values in these alpha diversity metrics in seawater, highlighting the contrasting ecological responses between the two habitats. Similar differences in bacterial species richness and diversity between sand and seawater were reported by Halliday et al. (2014) for beaches in California and Massachusetts sampled during the summer, as well as by Kostka et al. (2011) for a north Florida beach. This suggests a consistent pattern in coastal environments in which sand has a richer and more diverse bacterial community than seawater, and that the bacterial communities in the two habitats respond differently to environmental influences.

On a broad scale, our results also show similarities to previous studies in terms of the dominant bacterial taxa in coastal environments (Halliday et al. 2014; Wang et al. 2019, 2020; Ward et al. 2017), with Bacteroidota, Alphaproteobacteria, Actinomycetota, and Cyanobacteriota being the most prevalent groups of bacteria in sand and seawater throughout the 1‐year sampling period. While sand and seawater differed in their overall composition, patterns emerged in how proportions of these taxa in the community correlated with environmental parameters. In both habitats, the greatest proportions of sequences classified as Gammaproteobacteria and Bacteroidota, and the proportions of these groups in the community negatively correlated with temperature and DO. Both groups are common in the water column of marine systems, and can proliferate after algal blooms, when large inputs of dissolved organic matter (DOM) become available for heterotrophic microbes (Bunse and Pinhassi 2017; Fuhrman et al. 2015). Gammaproteobacteria and Bacteroidetes have also been identified as the most abundant bacterial taxa in intertidal sand samples from other coastal systems (Boehm et al. 2014; Halliday et al. 2014), including other parts of the northern Gulf of Mexico (Kostka et al. 2011; Newton et al. 2013). Thus, while bacterial communities in sand and seawater along the Mississippi coast differed in their overall composition and response to environmental conditions, the dominant phyla in these two habitats were similar and showed some consistency in their relationships to key environmental drivers.

At a finer taxonomic level, ASVs classified as Gammaproteobacteria were widespread in sand and seawater, whereas ASVs classified as Bacteroidota were more strongly associated with sand. Bacterial species within the order Flavobacteriales (Bacteroidota) have previously been reported at high abundances at California beaches (Boehm et al. 2014; Halliday et al. 2014), as well as the northern Gulf of Mexico (Kostka et al. 2011; Newton et al. 2013). Various members of this order produce hydrolytic extracellular enzymes to break down DOM, giving them a competitive advantage in marine systems post‐algal bloom (Bunse and Pinhassi 2017). Consistent with this, activities of β‐glucosidase and NAGase were positively correlated with the proportions of Bacteroidota in sand, supporting the idea of their role in hydrolyzing complex organic substrates such as carbon and nitrogen. The ASVs identified as taxa within the order Flavobacteriales (ASV 13 = Flavobacteriaceae, ASV 24 = *Arcticiflavibacter*, ASV 38 = *Flavirhabdus*, ASV 81 = *Sediminicola*, ASV 284 = *Gramella*) were especially prevalent in sand, while previous studies have reported these taxa at high abundances in seawater (Bunse and Pinhassi 2017; Fuhrman et al. 2015; Halliday et al. 2014). These Flavobacteriales ASVs showed spatial and temporal variation and had differing relationships with environmental parameters. ASV 13, ASV 24, and ASV 38 were negatively correlated with temperature and DO, with ASV 24 and ASV 38 also showing positive correlations with pH and salinity. In contrast, the percentage of ASV 81 in the sand bacterial community showed the opposite correlations with temperature, pH, and salinity. Even these closely related strains of Flavobacteriales likely differ in their ecological niches, responding to specific combinations of oxygen availability, temperature, pH, and salinity. Thus, structuring of coastal bacterial communities likely occurs not only at the phylum level but at the finer level of ASVs.

Studies of bacterial communities of sand on beaches are limited, and sandy beaches have traditionally been viewed as “ecological deserts” (McLachlan and Defeo 2017). The studies that have examined bacterial assemblages in coastal sands (Basha et al. 2025; Buchan et al. 2014; Cui et al. 2013; Fuhrman et al. 2015; Halliday et al. 2014; Newton et al. 2013) have reported the presence of other taxa that are consistent with the current study. Halliday et al. (2014) found sequences classified as Planctomycetota at higher proportions in sand than in seawater, and we found that the proportion of Planctomycetota in the sand community was twice that in seawater. Similarly, Cui et al. (2013) found that *Pseudoalteromonas* spp. (Gammaproteobacteria) were important taxa in beach sand on Oahu (Hawaii), and ASV 17, the third most abundant ASV in our sand samples, classified as that genus. Previous studies on beach sands have looked for faecal indicator bacteria (Cui et al. 2013; Halliday and Gast 2011; Staley and Sadowsky 2016), which can be transported into nearshore waters (Boehm et al. 2014; Russell et al. 2012). This issue is relevant on the Mississippi coast, where seawater at beaches is routinely monitored for *Enterococcus*, which can lead to advisories and beach closures. To our knowledge, the sand bacterial community on these beaches has not been previously characterised or considered as a potential source for faecal or indicator bacterial contamination of the surrounding water. No ASVs classifying as *Enterococcus* were detected in our ‘core’ microbiome of sand or seawater. The sand bacterial community did contain sequences classified as Bacillota (formerly Firmicutes; the phylum that includes *Enterococcus*), which accounted for 3.3% overall of the bacterial sequences recovered from sand, with higher relative abundances observed during the March, June, and September 2024 sampling periods. Several abundant ASVs were identified as non‐pathogenic members of this phylum, such as ASV 35 (*Salirhabdus* sp.), ASV 204 (*Planococcus* sp.), and ASV 139 (*Anaerobacillus* sp.), but showed no strong pattern of correlation with their abundance and environmental variables. Notably, phylum Bacillota was not an important component of the seawater bacterial community, and sequences taxonomically assigned as this phylum were generally not detected in seawater samples.

*Enterococcus* is typically monitored as an indicator organism rather than a pathogen, so other groups may be of concern for environmental monitoring. In our dataset, 21 ASVs classified as *Vibrio* spp. (Gammaproteobacteria), none of which were part of the core microbiome, and there was no single *Vibrio* ASV that was consistently dominant across all beaches or sampling months. Several of these ASVs occurred at higher relative abundances in seawater than in sand; for example, ASV 118 was 10 times more abundant in seawater (0.32% of the community) than in sand (0.03%). Some of these *Vibrio* ASVs also exhibited temporal and spatial variability. ASV 118 accounted for a greater percentage of the seawater community in late summer and winter at multiple beaches, while ASV 216 and ASV 616 showed peaks in their relative abundance in June 2024 at beach 6. These sporadic increases suggest that *Vibrio* occurrence may be linked to localised or seasonal conditions, highlighting the importance of considering the influence of environmental factors on natural bacterial communities, which could act as reservoirs of potentially pathogenic groups. Establishing baseline knowledge of coastal sand‐ and seawater‐associated bacterial communities provides context for understanding their natural variability, the environmental factors shaping them, and their potential responses to future disturbances. From an applied perspective, such information contributes to ongoing efforts to monitor and assess public health risks associated with microbial contamination of beaches.

Previous studies have shown that temperature and salinity are among the strongest drivers of bacterial community composition in seawater (Fuhrman et al. 2006; Wang et al. 2019; Wang et al. 2020). Our results align with those studies, with both salinity and temperature affecting the bacterial community composition of seawater. The effects of salinity were particularly important, with it explaining over twice the variation in seawater bacterial community composition than water temperature, even though our sampling period spanned an entire year, sampling in all four seasons. Salinity in the nearshore Mississippi Sound is lower than average for the Gulf of Mexico (Cambazoglu et al. 2017; Vinogradov et al. 2004), being subject to a variety of freshwater inputs. One of the most abundant ASVs (ASV 2) that we found in seawater was identified as a member of a group more commonly found in freshwater lakes (Sporichthyaceae, *hgcl clade*; Actinomycetota; Cruaud et al. 2020; Pearman et al. 2022), suggesting a large influence of freshwater on our study sites. This ASV was particularly abundant at the westernmost beaches (4, 5, and 6) in March 2024, June 2024, and March 2025, and at the easternmost beaches (13 and 14) in September 2024. This tracked with conditions of lower salinity, and those groups of beaches are close to freshwater inputs from Bay St. Louis (beaches 4, 5, and 6) or Biloxi Bay (beaches 13 and 14). Importantly, the percentage of ASV 2 in the seawater bacterial community was strongly correlated with β‐glucosidase activity, suggesting a potential role in organic matter degradation during times of nutrient‐loaded freshwater inputs into this system.

Percentages of other ASVs in the bacterial community also suggest that the seawater community restructures under different salinity conditions. ASVs 1 and 3 (taxonomically assigned to *Cyanobium*) and ASV 2 (Sporichthyaceae) showed similar correlations with environmental parameters, with their representation in the community being greater under higher temperatures, higher DO, and lower salinity levels. In contrast, higher percentages of ASV 7 (Rhodobacteraceae) and ASV 14 (*Litorimicrobium*) in the community were correlated with higher salinity levels and pH, and lower temperatures and DO. These opposing relationships to environmental parameters may reflect a dynamic previously observed in marine systems, where heterotrophic Rhodobacteraceae exploit DOM released during and after phytoplankton blooms (Wang et al. 2020; Wietz et al. 2021) and could explain the inverse relationship between proportions of Alphaproteobacteria and Cyanobacteria observed in our study. Phytoplankton blooms are frequent and important in parts of the northern Gulf of Mexico, a nutrient‐rich region subject to anthropogenic pollution from nitrogen‐rich agricultural runoff in the Mississippi River. The collapse of such algal blooms can channel large amounts of organic matter into heterotrophic pathways, driving increased microbial respiration and oxygen depletion (Howarth et al. 2011; Mills et al. 2008). Interestingly, higher percentages of ASV 16 (*Synechococcus CC9902*) in the seawater community occurred in September and December, different sampling months than seen for the ASVs (ASV 1, ASV 3) identified as *Cyanobium*. The relative abundance of ASV 16 was also more correlated with lower DO and higher salinity conditions. This suggests that different photosynthetic picocyanobacteria are important at different times of the year, with *Cyanobium* being more common in the summer, while ASV 16/*Synechococcus* may be more dominant during winter months along the Mississippi coast, a time of cooler temperatures, reduced freshwater inputs, and higher salinity (U.S. Geological Survey 2025).

## 5 Conclusion

Our results highlight the distinct ecological dynamics of bacterial communities in sand and seawater along beaches of the Mississippi coast. Sand consistently supported richer and more diverse assemblages than seawater. Seawater communities showed stronger seasonal structuring and were particularly shaped by changes in salinity, potentially reflecting the strong influence of freshwater inputs from Bay St. Louis and Biloxi Bay in this coastal system. The contrasting distributions of closely related ASVs suggest that niche partitioning, in both sand and seawater, enables functionally similar strains to persist under different temporal, spatial, or environmental conditions. This highlights the importance of continuous monitoring of coastal microbial communities for understanding ecosystem function, resilience, and potential risks to public health. By establishing baseline knowledge of these coastal microbial assemblages, our study underscores the importance of natural variability in shaping microbial communities of beaches along the Mississippi coast. Importantly, it offers a framework for assessing future ecological and public health impacts under scenarios of increased freshwater inputs, eutrophication, pollution, and climate‐driven change.

## Author Contributions

**Stephanie N. Vaughn:** conceptualization; methodology; data curation; investigation; visualisation; formal analysis; writing – original draft; writing – review and editing. **Jacqueline C. Pavlovsky:** conceptualization; methodology; data curation; investigation. **Colin R. Jackson:** conceptualization; methodology; data curation; investigation; funding acquisition; resources; writing – review and editing.

## Funding

This work was supported by the U.S. Department of the Treasury; Mississippi Department of Environmental Quality; Mississippi Based RESTORE Act Center of Excellence.

## Conflicts of Interest

The authors declare no conflicts of interest.

## Data Availability

The datasets generated and collected during the current study are openly available in the NCBI Sequence Reads Archive under BioProject ID numbers PRJNA1329298 and PRJNA1330254 and at the Gulf of Mexico Research Initiative Information and Data Cooperative (GRIIDC; www.griidc.org) under the Unique Dataset Identifier of M3.x111.000:0001.
